# Beyond the Graft: Optimizing Post-Transplant Care in Primary Sclerosing Cholangitis

**DOI:** 10.3390/jcm15093480

**Published:** 2026-05-01

**Authors:** Chiara Becchetti, Raffaella Viganò, Francesca Aprile, Miki Scaravaglio, Giovanni Vitale, Giovanni Perricone, Chiara Mazzarelli, Marcello Vangeli, Luca Saverio Belli, Marco Carbone, Maria Cristina Morelli

**Affiliations:** 1 Hepatology and Gastroenterology Unit, ASST Grande Ospedale Metropolitano Niguarda, 20121 Milan, Italym.scaravaglio@campus.unimib.it (M.S.); giovanni.perricone@ospedaleniguarda.it (G.P.);; 2 Graduate School for Health Sciences, University of Bern, 3001 Bern, Switzerland; 3 Department of Medicine and Surgery, University of Milano-Bicocca, 20900 Monza, Italy; 4 Cambridge Stem Cell Institute, University of Cambridge, Cambridge CB2 0AW, UK; 5 Internal Medicine Unit for the Treatment of Severe Organ Failure, IRCCS Azienda Ospedaliero-Universitaria di Bologna, 40138 Bologna, Italy

**Keywords:** liver transplantation, inflammatory bowel disease, gastrointestinal cancer

## Abstract

Primary sclerosing cholangitis (PSC) is a chronic cholestatic liver disease characterized by fibro-inflammatory lesions of the biliary tree. In the absence of available, effective medical therapies, many patients progress to liver failure, making PSC one of the leading indications for liver transplantation (LT), despite its rarity. While LT in PSC is associated with good overall short- and long-term survival, post-transplant outcomes are limited by recurrent PSC (rPSC), which affects up to one quarter of PSC recipients with a significant risk of graft loss and re-transplantation. The risk of rPSC reflects a complex interaction between donor and recipient factors including associated inflammatory bowel disease (IBD), and long-term exposure to immunosuppression. Therefore, post-transplant management requires an individualized multidisciplinary approach and tailored immunosuppressive regimens aimed at balancing the risk of rejection and rPSC with the risk of infection and malignancy. Optimal control of IBD has emerged as a key modifiable determinant of rPSC risk and post-transplant outcomes. In addition, patients with PSC, particularly PSC-IBD patients, carry a significantly increased risk of hepatobiliary and colorectal cancer. Importantly, this oncological risk persists after LT. Thus, long-term, structured cancer surveillance must remain an integral component of post-transplant care. Looking ahead, novel therapies targeting shared hepatic and intestinal fibro-inflammatory pathways are currently being investigated to modify disease activity in the pre-transplant setting. Future studies are needed to assess whether these agents might be applicable also in the post-transplant setting to improve long-term graft and patient survival.

## 1. Introduction

Primary sclerosing cholangitis (PSC) is a chronic progressive disorder characterized by fibro-inflammation of the intrahepatic and extrahepatic bile ducts, leading to stricture formation and chronic cholestasis, which can progress to end stage liver disease and hepatobiliary cancers (in particular, cholangiocarcinoma [CCA] and gallbladder cancer). Up to 80% of cases of PSC present with inflammatory bowel disease (IBD), in particular ulcerative colitis, which is associated with an increased risk of colorectal and hepatobiliary malignancies [1].

**Figure 1:**
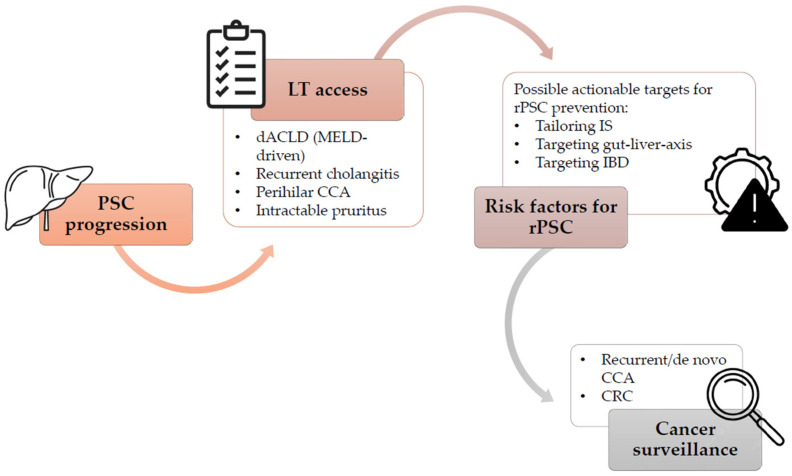
Flowchart of the clinical pathway starting from PSC progression and transplant listing, followed by post-transplant management. Abbreviations: PSC: primary sclerosing cholangitis; dACLD: decompensated advanced chronic liver disease; CCA: cholangiocarcinoma; rPSC: recurrent primary sclerosing cholangitis; IS: immunosuppression; IBD: inflammatory bowel disease.

Liver transplantation (LT) is currently the only curative option for patients with PSC and it is indicated in cases of end stage liver disease, debilitating biliary symptoms caused by strictures not amenable to endoscopic treatment, including intractable pruritus and recurrent or refractory acute cholangitis, and peripheral or hilar CCA (in the context of a clinical trial) [1,2,3]. Although overall long-term survival following LT in PSC is good with estimates of 93%, 87%, and 83% at 1, 5 and 10 years [2,3], challenges such as recurrent PSC (rPSC), high risk of acute cellular rejection (ACR) and IBD-related complications can significantly impact graft and patient post-LT outcomes (Figure 1).

rPSC is reported in 10–37% of patients who received a LT during a follow-up period of 5–10 years after LT, with the mean time to diagnosis of rPSC being 4.6 years after LT [1,2,3,4,5]. More recently a French experience with longer follow-up reported actuarial risk of developing rPSC at 5, 15, and 25 years of 15.6%, 37.9%, and 52.6%, respectively [6]. Factors associated with a higher risk of rPSC include recipient factors such as younger or older age at the time of LT, male sex, high model of end stage liver disease score, the presence of CCA, the presence of IBD, donor factors such as older donor age, sex mismatch, cytomegalovirus mismatch, and steroid-resistant rejection episodes [4,6]. In addition, both the presence and activity of IBD have been linked to rPSC, and efforts should be directed at maintaining IBD in remission [1,2]. rPSC is associated with a 4-fold increased risk of graft failure or death [1,2,3]. Therefore, efforts should focus on minimizing the risk of recurrence by optimizing IBD management, cancer surveillance and tailoring immunosuppressive therapy to balance the risk of graft failure and long-term mortality.

This review was conducted as a narrative review. The literature was primarily identified through iterative searches in PubMed and by screening the reference lists of relevant articles. Randomized controlled trials, systematic reviews, meta-analyses, and international guidelines were prioritized. Case reports with limited sample sizes, conference abstracts, and non–peer-reviewed gray literature were generally excluded unless they provided unique mechanistic insights.

## 2. Tailoring Immunosuppression in PSC After LT: Balancing Efficacy and Safety

The selection of immunosuppressive regimens should aim to minimize the pro-inflammatory state associated with the risk of rPSC. In particular, strategies aiming at the dual goals of preventing graft rejection while managing underlying IBD activity seems particularly promising. There are no standardized recommendations for post-LT immunosuppression in PSC. The optimal choice of calcineurin inhibitor (CNI) has been debated. Earlier studies suggested cyclosporine (CyA) might offer a lower recurrence rate but recent large-scale analyses focusing on long-term outcomes favor tacrolimus (TAC) [7].

A recent propensity-score matched analysis demonstrated that TAC, compared to CyA, provides significantly better overall patient and graft survival for PSC patients: respectively 72.8% versus 65.2% and 62.4% versus 53.8% at 10 years after LT [7]. TAC use was associated with a statistically significant reduced risk of graft loss or death. Based on these superior long-term results, TAC emerges as the preferred CNI and should be the standard therapy used after LT for PSC. In addition to the CNI type, the use of other common immunosuppressants was assessed in relation to the risk of graft loss or death. The use of mycophenolate (MMF) in combination with a CNI was associated with a reduced risk of graft loss or death, suggesting a beneficial, possibly immunomodulatory, effect of MMF on long-term survival, likely related to modulation of the underlying autoimmune response [8]. However, the impact of an immunosuppressive regimen including azathioprine (AZA), which can be considered also for the effect on the IBD maintenance of remission, adds another layer of complexity in managing PSC. Lastly, LT immunosuppression guidelines recommend a steroid-free approach except for specific groups, including patients with autoimmune liver disease [9]. This highlights the need for a steroid component, at least initially, to manage the autoimmune nature of PSC, even though a recent large-scale study finds steroid use to be significantly associated with the risk of rPSC [6].

Limited experiences have suggested that adding Rituximab (anti-CD20 B-cell depleting agent) to the immunosuppression regimen may delay or potentially prevent rPSC, based on the possibility of reducing rejection risk [10]. This approach stems from the idea that achieving operational tolerance may help minimize the risk of recurrence [11]. Along these lines, a single-center anecdotal report described the use of autologous hematopoietic stem cell transplantation in LT recipients with rPSC [12]. Two of the five patients achieved operational tolerance with no evidence of rPSC or ACR. One patient developed sinusoidal obstruction syndrome requiring re-transplantation with sirolimus monotherapy without evidence of rPSC. The remaining two patients died. Based on this experience, the authors concluded that although operational tolerance was achieved in some cases, the high morbidity associated with the procedure makes this specific protocol unsuitable as a clinical option. Overall, a triple-immunosuppression regimen consisting of TAC, an anti-proliferative agent, and corticosteroids appears to represent a reasonable strategy at the time of LT, followed by progressive steroid withdrawal and dual-agent maintenance therapy in the long term. However, a recent survey conducted by the International PSC Study Group demonstrated substantial heterogeneity across transplant centers in long-term immunosuppressive regimens, steroid withdrawal strategies, and target TAC exposure [13]. This variability translates into differences in cumulative immunosuppressive burden following LT with uncertain impact on post-LT outcomes, highlighting the lack of evidence-based standards and the need for comparative studies in PSC.

## 3. Emerging Biomarkers and Scores for Monitoring and Predicting rPSC

Despite the current standard for diagnosing rPSC is a combination of radiological findings and histological assessment in the absence of other causes (i.e., hepatic artery thrombosis or chronic rejection), clinicians are actively exploring several emerging, non-invasive biomarkers for immune monitoring related to the risk and presence of rPSC. Since multiple episodes of ACR are linked to rPSC, an early diagnosis of rejection is crucial. A summary of the possible non-invasive markers in rPSC is listed in Table 1, detailing not only the non-invasive markers related to early detection of ACR, but also microbiome-related biomarkers and molecular and cell-based biomarkers specifically targeting gut-liver axis and biliary epithelium, which seem particularly promising and specific. Besides non-invasive biochemical markers, two scores, the United-Kingdom (UK-PSC) and Amsterdam-Oxford (AOPSC) scores currently applied outside of the LT setting as prognostic models for PSC outcomes, were validated in cohort of 67 patients with rPSC. Both UK-PSC and AOPSC scores were independently associated with graft loss, with a threshold of ≥−4.2 and ≥2.4, respectively, but only the UK-PSC score was independently associated with overall mortality [14]. Considering the small simple size, caution is warranted regarding their generalizability of these findings, and further large-scale prospective studies are essential before these scores can be formally recommended for clinical decision-making.

## 4. Target Therapy and Future Perspective in rPSC

Pharmacological management of PSC remains a significant challenge, especially in the post-LT setting where recurrence (rPSC) affects between 10% and 37% of grafts [1,2,3,4,5]. While no approved disease-modifying therapy exists, several novel drug classes are currently under investigation in the native liver with potential implications for managing or preventing recurrence [15]. Biliary and nuclear receptors are among the main targets. Standard ursodeoxycholic acid (UDCA) continues to be used at doses of 13–23 mg/kg/day, though meta-analyses have shown no significant effect on transplant-free survival [16], and its use as a preventive treatment post-LT has not shown an impact on the incidence of rPSC [6,17]. Nor-Ursodeoxycholic acid (NCA), a synthetic side-chain-shortened analogue of ursodeoxycholic acid, represents a novel approach to bile acid modulation, that promotes biliary tree choleresis and prevents the build-up of toxic bile acids [18]. Results from a pivotal Phase III trial in native PSC demonstrated that NCA achieved partial normalization of alkaline phosphatase (ALP) and prevented histological disease progression over two years [19]. While specific clinical trials for recurrence are pending, its flushing mechanism makes it a strong candidate for future rPSC prevention. In the Peroxisome Proliferator-Activated Receptors (PPAR) Agonists category there are highly promising drugs thanks to their ability to downregulate bile acid synthesis and promote choleresis [20]. Elafibranor (a dual alpha/delta agonist) showed significant biochemical improvement, including ALP reduction and pruritus relief, in the 12-week Phase II ELMWOOD trial for native PSC [21] and a Phase III trial is already started. Bezafibrate (a pan-PPAR agonist) has demonstrated superiority over placebo for ameliorating pruritus in the FITCH trial [22]. A Phase IV trial (NCT06905054) is currently investigating whether daily fenofibrate can prevent clinically detectable rPSC over 36 months, testing the hypothesis that mitigating cholestasis can impede biliary injury in the allograft. Despite the recent clinical setbacks and withdrawal of obeticholic acid, farnesoid X receptor remains a relevant theoretical therapeutic target for preventing rPSC. Current research is shifting toward next-generation non-steroidal agonists, such as Cilofexor, aim to improve tolerability, however results on their safety profile remain unconvincing [23], making these drugs unsuitable for the complex setting of post LT. Recent research has shifted toward directly targeting pathways driving peribiliary fibrosis [15]. Nebokitug (CM-101) is a monoclonal antibody that neutralizes the chemokine CCL24, which recruits immune cells and activates fibroblasts. In the Phase II SPRING trial, nebokitug demonstrated significant anti-fibrotic activity, particularly in patients with moderate to advanced disease [24]. Similarly, Bexotegrast, an oral dual-selective inhibitor of alpha vs. beta6 and alpha vs. beta1 integrins, stabilized markers of liver fibrosis and improved bile flow in Phase IIa trials [25]. While these agents show disease-modifying potential, clinical evidence in the transplant setting is still lacking. In the specific context of rPSC, oral vancomycin has been documented in case reports to achieve complete biochemical normalization in both pediatric and adult patients [26,27]. Currently a phase II trial in native liver is ongoing [28]. Recent preclinical studies have identified Claudin-1 as a potential therapeutic target in PSC. Monoclonal antibodies against Claudin-1 reduced fibrosis, cholestasis, and the ductular reaction, supporting their potential relevance for biliary disease drug development [29].

**Figure 2:**
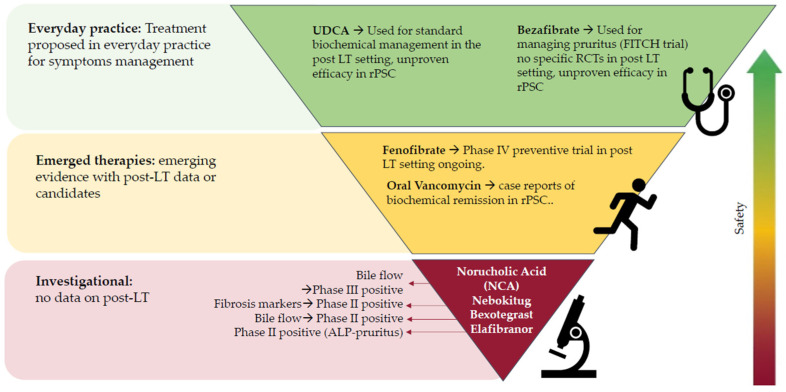
Developing medications in PSC and possible implication for rPSC. Abbreviations: rPSC: recurrent primary sclerosing cholangitis; UDCA: ursodeoxycholic acid; NCA: Nor-Ursodeoxycholic acid; ALP: alkaline phosphatase.

Despite progress in PSC drug development, clinical use in the post-LT setting requires careful management of interactions with immunosuppressants, highlighting the need for dedicated trials to establish long-term safety and efficacy (Figure 2).

## 5. The Gut-Liver Axis: Role of Microbiota in Post-Transplant Outcomes

PSC, differently from healthy controls, is associated with specific alterations of the gut microbiota that contributed to the definition of a PSC-associated microbial profile [30]. Common findings include reduced alpha diversity (i.e., species variability among different samples or sites), enrichment of genera such as Veillonella, Enterococcus, Klebsiella, and Escherichia and reduction of others including Lachnospiraceae and Faecalibacterium [31,32,33]. Importantly, these alterations seem to be consistent across different geographical regions [34], and most studies suggest they are independent from IBD status [31,32,35] and persist after LT [33]. The gut microbiota composition in the post-LT PSC population is highly different from healthy controls, but similar to pre-LT PSC [33]. This contrasts with other chronic liver diseases where LT often results in marked changes towards a more “normalized” microbial profile [36]. Between 15 and 30% of individuals develop rPSC after LT, which significantly impacts both graft and patient survival. Comparative profiling of rPSC and non-rPSC revealed similar alpha, but greater beta diversity in rPSC (i.e., species variability among different samples or sites). Five microbial alterations were associated with PSC and rPSC compared with healthy controls, including reduction in the genera Lachnospiraceae ND3007, Lachnospiraceae CAG-56, and Bilophila and increase in genera Streptococcus and Hungatella [33]. However, available data on post-LT PSC associated microbiota is still limited, comes only from mucosal and not from faecal microbiota and requires further validation.

The impact of IBD on PSC-associated dysbiosis has been investigated in both faecal and mucosal microbiota studies. Most evidence indicates that faecal microbiota profiles do not differ significantly between PSC patients with and without IBD, supporting the concept that dysbiosis is primarily driven by the liver disease itself [32,35,37,38]. In line with this, one mucosal microbiota study reported no differences in alpha diversity between PSC patients with and without IBD, irrespective of LT status. However, when analyzed separately, PSC-LT patients with IBD showed lower alpha diversity in the ascending colon compared with those without IBD. In addition, the same study found reduced relative abundance of Akkermansia across all colonic segments in PSC-IBD. As these findings are derived from a single study, validation in larger patient cohorts is necessary.

The clinical implications of PSC-associated microbial dysbiosis have been investigated. Data from a cross-sectional study demonstrated that a dysbiosis index derived from the five PSC-associated genera and the presence of the potential pathobiont Klebsiella, was associated with clinical outcomes. Specifically, higher index values were associated with reduced transplant-free survival (aHR 1.28, 95% CI 1.02–1.61; *p* = 0.03, adjusted for Mayo risk score) and showed a trend toward worse recurrence-free survival [38]. Beyond microbial composition, dysbiosis related alterations in circulating metabolites, and in particular vitamin B6 [38,39] were found associated with clinical outcomes before and after LT. Longitudinal validation of these findings will support the use of PSC-associated microbial and metabolic abnormalities as surrogate markers of LT- and recurrence-free survival as well as potential therapeutic targets. Diet is a key modulator of the gut microbiota and may influence the gut–liver axis in PSC. Dietary patterns have been shown to affect microbial diversity, bile acid composition, and the production of microbial metabolites, all of which are implicated in liver inflammation and fibrogenesis [40]. In particular, fiber rich diets have been associated with increased abundance of short-chain fatty acid-producing bacteria, whereas diets enriched in fat and processed foods may promote expansion of pathobionts and alter bile acid pools [41,42,43]. These mechanisms provide a biological rationale for dietary modulation as a potential therapeutic strategy. However, evidence in PSC remains limited and largely observational, with no controlled studies demonstrating a clear clinical benefit [44]. At present, no specific dietary intervention can be recommended to modify disease course, and nutritional approaches should be considered supportive rather than disease-modifying. Although definitive causal proof is challenging in humans, collectively there is enough evidence to support the rationale of targeting the gut microbiota to modify the progression of liver disease in PSC. Interventions such as dietary modifications, antibiotics, and faecal microbiota transplantation (FMT) have been proposed both to halt the progression of PSC and to prevent recurrence after LT. Among antibiotics, oral vancomycin has shown the most promising results while maintaining a favorable safety profile. Case series and small trials have reported improvements in liver biochemistry, histology, and even reversal of early disease before and after LT [45,46]. However, the optimal dosing and duration of oral vancomycin are unknown, and large, long-term studies are required to evaluate its sustained efficacy and safety.

Beyond antibiotics, FMT has emerged as a strategy to restore microbial diversity and reduce pathobionts. In PSC, pilot studies have demonstrated biochemical improvement and increased microbial diversity following FMT from healthy donors [47]. As an alternative therapeutic tool for targeting specific genera within the PSC-associated gut microbial profile, increasing attention has been paid to the use of bacteriophages, self-replicating viruses that infect bacteria with high host specificity. Ichikawa and colleagues [48] generated a bacteriophage cocktail specifically targeting a K. pneumoniae strain (derived from a patient with PSC) and successfully demonstrated its efficacy in a DDC-fed murine model of hepatobiliary injury.

At present, there are no guideline-recommended microbiota-directed or nutrition-based disease-modifying therapies for PSC, including in the post-transplant setting. Oral vancomycin and FMT should not be used routinely in clinical practice outside specialized centers or clinical trials, given the limited evidence base.

Overall, PSC-associated microbiota represents a promising prognostic marker and therapeutic target. However, the current evidence base is largely exploratory, with most data derived from small, single-centre, observational or preclinical studies. As such, these findings should be interpreted cautiously and do not yet support routine clinical implementation. Ongoing trials (NCT05876182 and NCT06286709) will clarify long-term efficacy and safety [49], but patients awaiting or after LT remain largely excluded, leaving a major evidence gap.

## 6. Managing Coexisting IBD in the Post-LT Setting

PSC and IBD frequently coexist, and large registry data indicate that approximately two-thirds of patients with PSC listed for LT have concomitant IBD [8], with the inflammatory activity of the bowel potentially acting as a key driver that modulates the progression of the liver disease. While IBD is present in up to 80% of the general PSC population, its prevalence is slightly lower (approximately 63–66%) in the specific subgroup of patients undergoing LT. [50]. The effect of LT on IBD course has been evaluated in both comparative cohorts and meta-analyses. In the multicenter LIVIBD study, PSC-IBD patients undergoing LT were compared with non-transplanted PSC-IBD controls, and LT was not identified as an independent risk factor for a more severe IBD course, including need for escalation therapy, hospitalization, or colectomy [51]. However, in the ECCO-JCC systematic review and meta-analysis focused specifically on PSC-IBD outcomes after LT, 17.8% of patients with concomitant IBD at transplantation experienced a clinical exacerbation during follow-up [52]. Meta-regression analyses demonstrated a significant temporal decline in exacerbation rates over calendar years, a finding limited to Eurpean cohorts, suggesting improvements in immunosuppressive strategies and post-transplant monitoring [52].

Overall, LT does not uniformly induce intestinal remission, and post-transplant IBD activity appears to be more strongly influenced by the immunosuppressive regimen than by transplantation itself [53,54,55]. Across published cohorts, approximately 30–50% of patients experience either worsening of pre-existing IBD or de novo disease after LT, whereas sustained remission is reported in only a minority, often less than 20–25% of cases [54,56]. These observations highlight that post-transplant IBD remains an active disease entity requiring dedicated management.

Immunosuppressive choice plays a pivotal role in defining post-transplant IBD phenotype. Earlier observational studies suggested higher rates of IBD flares and steroid dependency with TAC compared with CyA, with flare rates exceeding 40% in TAC-based regimens versus approximately 20–25% in CyA-treated patients [54,55,57]. However, more recent propensity-matched analyses from Europe did not confirm a clear long-term superiority of CyA in terms of IBD-related outcomes, hospitalization, or colectomy rates [7].

AZA-containing regimens have consistently been associated with a more stable colitis phenotype, with lower reported colectomy rates (often <15–20%) compared with AZA-free regimens [54,58]. Maintenance with CyA and/or AZA has been associated with a higher proportion of quiescent disease compared with TAC-dominant strategies [56,58]. Thus, optimization of immunosuppression requires careful balancing of intestinal disease control, graft protection, and rPSC risk.

Despite optimized antirejection regimens, a substantial proportion of transplanted PSC-IBD patients remain symptomatic and require escalation to IBD-directed therapies, including biologics and small molecules [56,59]. Current evidence supports an individualized immunosuppressive strategy. While CyA and AZA remain reasonable options in patients with severe colitis, CyA cannot be considered a universal first-line calcineurin inhibitor, given its higher risk of ACR compared with TAC [7,60].

### 6.1. Biologic Therapy

Biologic and targeted therapies appear effective for intestinal disease control and generally safe when combined with standard antirejection regimens [61]. Vedolizumab is the most extensively studied agent in this setting. Case series in moderate-to-severe post-LT IBD, including PSC-IBD, report clinical response rates ranging from 40% to 70% and endoscopic improvement in approximately 30–50% of patients, with low rates of serious adverse events [62]. A systematic review in LT recipients confirmed effective post-transplant IBD control with vedolizumab and reported no excess risk of rejection or malignancy [63]. Anti-tumor necrosis factor (TNF) agents, often used as historical comparators, achieve similar rates of clinical remission and steroid-free response in more recent cohorts, with no consistent signal of increased ACR or graft loss [64]. Multiple cohorts and a systematic review/meta-analysis support their efficacy and acceptable safety profile in post-LT IBD [59,64]. Emerging data support the use of ustekinumab and Janus kinase (JAK) inhibitors. In a multinational PSC-IBD LT cohort, ustekinumab achieved remission rates comparable to vedolizumab with a similar safety profile [64,65]. Systematic reviews and dedicated LT series suggest that JAK inhibitors provide clinically meaningful benefit with acceptable safety, although class-specific risks mandate careful monitoring [66,67]. Evidence for IL-23–targeted therapies in PSC-IBD after LT is currently lacking, with only anecdotal data in LT recipients without PSC-IBD–specific outcomes [68]. While these findings remain preliminary, they suggest a potential translational pathway for IL-23-targeted therapies and JAK inhibitors; however, their establishment as standard of care with a sufficient level of recommendation awaits validation through larger, multi-center prospective studies.

From a safety perspective, no consistent increase in ACR has been attributed to biologics or small molecules in LT recipients. Infections remain the principal concern and reflect cumulative immunosuppression rather than drug-specific toxicity. Data on JAK inhibitors highlight the need for vigilance regarding serious infections and thrombotic events; however, such events have not been confirmed in the reported post-LT experience [66,67] (Table 2).

### 6.2. Colectomy

Despite effective medical therapy, colectomy remains common in PSC-IBD. In a retrospective series of 127 PSC-IBD patients undergoing total abdominal colectomy with or without LT, long-term survival after LT was similar regardless of surgical sequence, whereas post-colectomy survival was significantly shorter when colectomy followed LT (median 16.0 vs. 42.6 years, *p* = 0.007) [69]. Medically refractory disease was associated with higher rates of rPSC and biliary complications compared with oncologic indications [69]. Pouch-related outcomes are also relevant: a large single-center cohort reported chronic pouchitis rates of 67% in transplanted patients versus 45.5% in non-transplanted controls (*p* = 0.03), without differences in overall or pouch survival [70]. A meta-analysis confirmed a pooled pouchitis risk of 65% in PSC-IBD patients with LT, increasing to 83% when the intervention preceded LT (odds ratio significantly higher compared with LT-first strategies, *p* < 0.001), reinforcing that PSC-IBD itself, rather than LT, is the dominant driver of pouch inflammation [52].

## 7. Cancer Surveillance: A Pillar of Long-Term Follow-Up

PSC remains highly premalignant after LT, with excess risk of colo-rectal cancer (CRC) in patients with IBD, de novo or recurrent hepatobiliary malignancies including CCA, increased lymphoma risk, and post-LT cancers from chronic immunosuppression [71,72,73]. LT corrects hepatic failure but does not “reset” the underlying oncogenic diathesis, justifying life-long oncologic surveillance after LT [72,74]. In PSC cohorts, de novo malignancies are shifted toward gastrointestinal, hepato-pancreatobiliary tumors and lymphoid neoplasms, reflecting interaction between intrinsic PSC-IBD oncologic phenotype and long-term immunosuppression [73,74]. In fact, the persistent inflammatory stimulus of IBD acts as a potent driver for colorectal carcinogenesis, while systemic immunosuppression further weakens the immune surveillance against both hepatobiliary and extra-hepatic cancers.

De novo cancer incidence after LT for PSC is comparable to or slightly higher than non-PSC LT cohorts, with a pattern skewed toward gastrointestinal and hematologic malignancies, including post-transplant lymphoproliferative disorders (PTLD) [73]. Large registry analyses confirm malignancy contributes substantially to late mortality despite 10-year survival exceeding 80% [71,75]. PSC is associated with threefold higher lymphoma risk (HR 3.0, 95% CI 1.6–5.7) [72]. Post LT, PSC recipients share increased PTLD risk, with lymphoproliferative diseases among the most frequent de novo malignancies, significantly reducing post-LT survival [73]. Given the elevated risk of lymphoma and other de novo malignancies under immunosuppression, surveillance strategies should explicitly incorporate regular clinical assessment for “B symptoms” or lymphadenopathy, vigilance for unexplained cytopenias, and prompt investigation of any suspicious systemic features, particularly in Epstein-Barr Virus seronegative recipients or those receiving intensive immunosuppressive regimen [73]. Finally, PSC recipients should follow general post LT cancer screening strategies, including regular dermatologic examinations for skin cancer, counselling on sun protection, and age and sex appropriate screening for solid tumors such as breast, cervical, prostate and lung cancer, with a tendency toward a proactive approach given the cumulative impact of lifelong immunosuppression [76,77].

### 7.1. Recurrent/De Novo CCA

Post-LT CCA in PSC is rare but has poor prognosis; surveillance must integrate rPSC risk [73,78,79]. In PSC recipients without pre-LT CCA, rPSC, complex strictures, repeated cholangitis, severe IBD, and older donor age support intensive surveillance [80,81]. Surveillance is risk adapted: lifelong biliary imaging (magnetic resonance or ultrasound) at regular intervals, with shorter intervals and low threshold for endoscopic retrograde cholangiopancreatography and biopsy in rPSC or new cholestasis [82]. In patients transplanted for perihilar CCA in PSC, recurrence risk relates to pre-transplant tumor burden. The PRETREAT score stratifies CCA recurrence risk using four predictors: macroscopic residual tumor, vascular encasement, lympho-vascular invasion, and radial tumor diameter. Patients were stratified into low, moderate and high-risk groups with 5-year recurrence-free survival of 89.0%, 38.3%, and 15.4%, respectively (*p* < 0.001). Overall survival improved only in moderate-risk patients (70.4% vs. 46.9%, *p* = 0.024). This score can serve to define the frequency of imaging during the first 2–5 years post LT, when the risk of recurrence is highest (e.g., cross-sectional imaging every 3–6 months for high score patients) [78].

### 7.2. CRC

PSC-IBD confers 4-fold higher CRC risk versus IBD alone and 10-fold higher risk than the general population [83]. Overall cumulative risk of colorectal neoplasia from IBD diagnosis was 3.3%, 6.4%, 17%, and 25% at 5, 10, 20, and 25 years, with most lesions arising in the proximal colon [83]. A 2013 meta-analysis found post-LT CRC incidence of 5.8/1000 person-years in PSC and 13.5/1000 person-years in PSC-IBD, markedly higher than unselected LT recipients (around 1.2/1000 person-years). Longer IBD duration and extensive colitis increased post-LT CRC risk, whereas the immunosuppression type was not clearly linked [54]. LT does not abolish CRC risk in PSC-ulcerative colitis, but low-grade dysplasia (LGD) after LT may carry lower progression risk than LGD before LT [84]. Proposed mechanisms include more frequent inactive colitis in LT recipients, systemic immunosuppression, and reduced exposure to carcinogenic bile acids after removal of the affected liver. In a multicenter cohort of 320 PSC-IBD patients with LT vs. 659 without, 21% vs. 26% developed CRC; LT was associated with reduced risk (aOR 0.66, 95% CI 0.47–0.93) [85]. Despite this, one-fifth of LT patients developed neoplasia, supporting LT as risk-modifying rather than risk-negating. Most cohorts and series do not systematically evaluate CRC, and it is rarely a predefined endpoint, although the long-standing CRC risk inherent to PSC IBD is repeatedly acknowledged [63,86,87]. In the nationwide PSC IBD LT anti TNF series, particular concern is raised regarding CRC risk, with a strong recommendation for intensive colonoscopy surveillance rather than attributing risk directly to biologic exposure [86].

Pooled LT data with vedolizumab do not show a clear excess of overall malignancy, but follow up is limited and numbers remain small, precluding firm conclusions [63]. Given the intrinsic oncologic risk in PSC IBD and the background of chronic immunosuppression after LT, long term pharmacovigilance is warranted irrespective of the specific biologic or small molecule agent used.

PSC-IBD patients, including post-LT, should remain in intensive colonoscopy surveillance as absolute CRC risk remains high. Current recommendations support annual colonoscopy starting 8–10 years after diagnosis in ulcerative/Crohn’s colitis, and from PSC onset in PSC-IBD, continuing yearly. Additionally, guidelines recommend life-long CRC surveillance in PSC-IBD after LT without de-escalation [72,74]. High quality colonoscopy with extensive biopsies (or chromoendoscopy) at 1–2 years intervals is advised from PSC-IBD diagnosis, maintained post LT; proctocolectomy remains an option for multifocal low grade or high-grade dysplasia [76,88].

## 8. Conclusions

**Figure 3:**
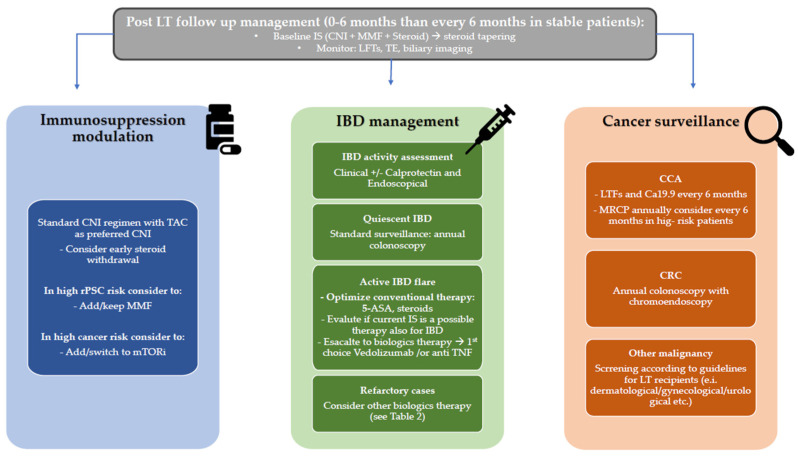
Integrated practical management algorithm for PSC-IBD patients post-LT. Abbreviations: 5-ASA: 5-aminosalicylic acid (mesalazine); CCA: cholangiocarcinoma; CRC: colo-rectal cancer; CNI: calcineurin inhibitor; IBD: inflammatory bowel disease; IS: immunosuppression; LFTs: liver tests of function LT: liver transplantation; MMF: mycophenolate mophetil; mTORi: mammalian target of rapamycin inhibitor; PSC: primary sclerosing cholangitis: rPSC: recurrent primary sclerosing cholangitis TAC: tacrolimus; TNF: tumor necrosis factor.

Although PSC is a rare disease, it accounts for a disproportionate burden of LT activity worldwide, reflecting a complex interplay between recurrent cholangiopathy, associated intestinal inflammation, long-term immunosuppression and increase cancer risk. Post-transplant outcomes are generally favorable; however, they are limited by a substantial risk of disease recurrence, which remains one of the main determinants of long-term survival and graft failure. In this complex post-LT setting, the optimal management of IBD and the careful tailoring of immunosuppressive regimens are central to improving outcomes. In addition, cancer surveillance represents another fundamental aspect to consider in order to improve overall patient survival, beyond graft survival (Figure 3).

The emergence of novel pharmacological targets acting on both hepatic and intestinal pathways represents a major advancement and further supports a multidisciplinary approach of post-transplant care in PSC. A major challenge remains the scarcity of high-quality evidence, as most current management strategies are based on retrospective data rather than prospective clinical trials specifically designed for the PSC-IBD post-transplant setting. Future research must aim to consider this sub-set of patients in large, prospective multicenter studies and bridge the gap between bench and bedside, ensuring that emerging insights into the gut-liver axis and novel molecular biomarkers achieve a meaningful translation into routine clinical practice for better risk stratification.

## Figures and Tables

**Table 1:** Biomarkers for monitoring of rPSC after LT.

Category	Biomarker	Utility and Level of Evidence	Clinical Applicability
**Standard Care**	ALP (Alkaline Phosphatase)	**Clinical Practice.** The primary biochemical marker for disease activity and response to therapy.	Monitoring/Prognosis
**Surveillance**	Ca19.9	**Clinical Practice.** Used for Cholangiocarcinoma surveillance, despite limitations in specificity.	Screening cholangiocarcinoma
**Fibrosis**	ELF Score/Transient elastography	**Clinical & Research.** Non-invasive assessment of liver stiffness and predictive of liver related outcomes.	Prognosis/Monitoring
**Rejection**	Circulating donor-derived cell-free DNA (dd-cfDNA)	**Research/Emerging.** Early detection of rejection, before alterations in liver enzymes with high sensitivity and negative predictive value for detecting graft injury.	Monitoring
	Preformed and de novo donor specific antibodies (DSAs)	**Clinical Practice.** Particularly against class II HLA antigens, have been associated with graft loss, chronic rejection, and biliary complications.	Prognosis/Monitoring
	Specific microRNAs (e.g., miR-122, miR-155, miR-181a-5p)	**Research.** Associated with rejection and immune activation. Their use in everyday clinical practice remains limited.	Monitoring
**Gut-Liver Axis**	Specific bacterial taxa	**Research.** Patients without recurrence were characterized by an increased presence of Gammaproteobacteria Shigella Expansion of Proteobacteria is more pronounced in PSC patients after LT.	Prognosis/Research
	Intestinal bacteria and genetics	**Research.** Bacterial translocation is associated with rPSC. This risk is highest when combined with the FUT2 non-secretor status, a genetic risk factor that influences the gut mucosal barrier and is associated with rPSC.	Prognosis
**Immuno-serology**	IgA Anti-Glycoprotein 2 (GP2) Antibodies	**Research.** Identifies patients with a more severe disease phenotype and poor survival in PSC relevant in recurrence risk.	Prognosis
	Atypical p-Anti-Neutrophil Cytoplasmic Antibodies (ANCAs)	Common in PSC. The presence of PSC-related HLA alleles associated with ANCA formation may also be linked to an increased risk of ACR, a risk factor for rPSC	Diagnosis/Prognosis
	Soluble Vascular Adhesion Protein-1 (sVAP-1)	**Research.** Elevated sVAP-1 levels are associated with adverse disease outcomes in PSC, as it correlates with T-cell homing from the gut to the hepatobiliary tract	Prognosis
**Molecular/Cell**	Biliary epithelial cell senescence	**Research.** These cells under stress produce inflammatory mediators (SASP), which promote inflammation and fibrosis. Markers of cholangiocyte injury and senescence are under investigation.	Research
	Mucosal-Associated Invariant T (MAIT) cells	**Research.** MAIT cells are enriched in the liver and recognize microbial-derived metabolites. Bile from PSC patients can contain antigens that activate MAIT cells, suggesting a direct pathophysiological link between the biliary microbiome and the immune system that could drive rPSC.	Research
	Cell-free DNA (cfDNA)	**Research.** Besides its application for ACR or cancer recurrence, the concept of cfDNA is being explored to detect graft injury or inflammation specifically related to rPSC.	Monitoring

**Table 2:** Maintenance therapy in IBD-PSC post LT with level of evidence in its use.

Class/ Molecule	Mechanism of Action	Pro (Post-LT Context)	Cons (Post-LT Context)	Use Status
**Vedolizumab** (monoclonal antibody)	Targets alfa4beta7 integrin; selectively inhibits lymphocyte trafficking to gut (gut-specific immunomodulation)	Sustained intestinal efficacy; favorable safety profile; no evidence of increased acute rejection or malignancies	Limited data on numbers/follow-up; no improvement in liver biochemistry or PSC course	**OK**
**Anti-TNF agents** (monoclonal antibodies)	Systemic TNF-alfa neutralization (key cytokine in gut-liver axis)	Effective post-LT IBD control; acceptable graft outcome; no increased risk of rejection rates	Systemic immunomodulatory effect; comparative studies limited by non-randomized design.	**OK**
**Ustekinumab** (monoclonal antibody)	Targets p40 subunit (IL-12 and IL-23) inhibits Th1 and Th17 pathways	Clinical remission and safety profile comparable to vedolizumab; no consistent signal of increased rejection	Systemic mechanism of action requires careful monitoring in LT setting	**OK**
**JAK inhibitors** (e.g., Tofacitinib, Upadacitinib)	Inhibition of intracellular cytokine signaling (multiple pro-inflammatory pathways)	Clinically meaningful IBD control in selected pot-LT patients	Theoretically concerns for serious infections thromboembolic events drug-drug interactions	**CAUTION**
**Selective IL-23 p19 inhibitors** (e.g., Risankizumab, Mirikizumab)	Selective inhibition of Th17-mediated inflammation (spares IL-12)	Theoretical safety advantage; once case report (post-LT psoriasis) shows good tolerability without graft dysfunction	Lack of evidence in post-LT PSC IBD; currently considered investigational option	**INVESTIGATIONAL/** **UNKOWN**
**Azathioprine** (Thiopurine/Antimetabolite)	Inhibits purine synthesis, suppressing immune cell proliferation B and T	Established historical use in LT and IBD; oral administration	Risk of myelosuppression (leukopenia) and hepatotoxicity; increased risk of skin cancer	**CAUTION/MONITORING**
**Cyclosporine** (CNI)	Inhibits calcineurin, suppressing T cell activation and IL-2 production	Often already part of anti-rejection regimen, rapid effect as rescue therapy in severe colitis	Narrow therapeutic index; significant nephrotoxicity, hypertension, neurotoxicity, less used for long term IBD maintenance	**LESS PREFERABLE** **/MONITORING**

Abbreviations: CNI: calcineurin inhibitor; IBD: inflammatory bowel disease; LT liver transplantation; PSC: primary sclerosing cholangitis: TNF: tumor necrosis factor.

## Data Availability

No new data were created or analyzed in this study. Data sharing is not applicable to this article.

## Abbreviations

The following abbreviations are used in this manuscript: ACR: acute cellular rejection; PSC: primary sclerosing cholangitis; IBD: inflammatory bowel disease; rPSC: recurrent primary sclerosing cholangitis; CCA: cholangiocarcinoma; LT: liver transplantation; CNI: calcineurin inhibitor; TAC: Tacrolimus; CyA: cyclosporine; MMF: mycophenolate mofetil; AZA: azathioprine; FMT faecal microbiota transplantation; UDCA: ursodeoxycholic acid; NCA: Nor-Ursodeoxycholic acid; ALP: alkaline phosphatase; PPAR: Peroxisome Proliferator-Activated Receptors; TNF: tumor necrosis factor; JAK: Janus kinase; PTLD: post-transplant lymphoproliferative disorders; LGD: low-grade dysplasia; CRC: colorectal cancer.
